# The impact and relevance of techniques and fluids on lung injury in machine perfusion of lungs

**DOI:** 10.3389/fimmu.2024.1358153

**Published:** 2024-03-06

**Authors:** Florian Ponholzer, Julia Dumfarth, Christoph Krapf, Andreas Pircher, Theresa Hautz, Dominik Wolf, Florian Augustin, Stefan Schneeberger

**Affiliations:** ^1^ Department of Visceral, Transplant and Thoracic Surgery, Center of Operative Medicine, Medical University of Innsbruck, Innsbruck, Austria; ^2^ Department of Cardiac Surgery, Medical University of Innsbruck, Innsbruck, Austria; ^3^ Department of Haematology and Oncology, Internal Medicine V, Medical University of Innsbruck, Innsbruck, Austria

**Keywords:** EVLP, PGD, ex vivo, organ perfusion, transplantation, techniques

## Abstract

Primary graft dysfunction (PGD) is a common complication after lung transplantation. A plethora of contributing factors are known and assessment of donor lung function prior to organ retrieval is mandatory for determination of lung quality. Specialized centers increasingly perform ex vivo lung perfusion (EVLP) to further assess lung functionality and improve and extend lung preservation with the aim to increase lung utilization. EVLP can be performed following different protocols. The impact of the individual EVLP parameters on PGD development, organ function and postoperative outcome remains to be fully investigated. The variables relate to the engineering and function of the respective perfusion devices, such as the type of pump used, functional, like ventilation modes or physiological (e.g. perfusion solutions). This review reflects on the individual technical and fluid components relevant to EVLP and their respective impact on inflammatory response and outcome. We discuss key components of EVLP protocols and options for further improvement of EVLP in regard to PGD. This review offers an overview of available options for centers establishing an EVLP program and for researchers looking for ways to adapt existing protocols.

## 1 Introduction

End-Stage Lung Disease (ESLD) often requires lung transplantation (LuTx) as a last resort for treatment. Despite the efforts to supply the demand, mortality on the waiting list remains a challenge (1). A possible countermeasure to this problem is the utilization of extended criteria donors (ECD). However, ECD lungs pose the risk of higher primary graft dysfunction (PGD). Extended criteria donor lungs have significantly higher early PGD 3 and lower intraoperative extracorporeal membrane oxygenation weaning rates in comparison to standard criteria donor lungs. However, survival and bronchiolitis obliterans syndrome rates do not differ (2, 3). To evaluate ECD lungs, pioneering centers established ex vivo lung perfusion (EVLP) as a means to assess organ quality. EVLP is a complex and multidimensional technology. The various individual specifications of the hardware, software and perfusate such as the type of pump, the ventilation modes or the composition of the perfusion solutions are evolving. While the core functions of EVLP have been well established, the evolution of this technology has only just started and a great many aspects remain to be advanced in order to eventually support stable multi-day long lung preservations suitable for organ regeneration and repair. Further to the immediate benefit for organ transplantation, EVLP may also serve as a research platform for primary or secondary lung cancer as described by Slama et al. (4) In order to create a meaningful platform for lung cancer research, EVLP might need to be prolonged to several days mimicking a physiological setting. First steps in this direction have been taken by Ali et al., who performed successful 3-day lung preservation utilizing a a cyclic normothermic ex vivo lung perfusion strategy. This strategy involved initial 6 hours of cold static storage at 4°C, followed by cold static storage at 10°C with two cycles of EVLP in-between (5). In liver perfusion models, extension of perfusion times allows for testing of new therapies *ex vivo* and thereby accelerate their development and eventual clinical use (6). This may add a valuable translational model system to the current armamentarium and help reduce animal testing.

**Figure 1:**
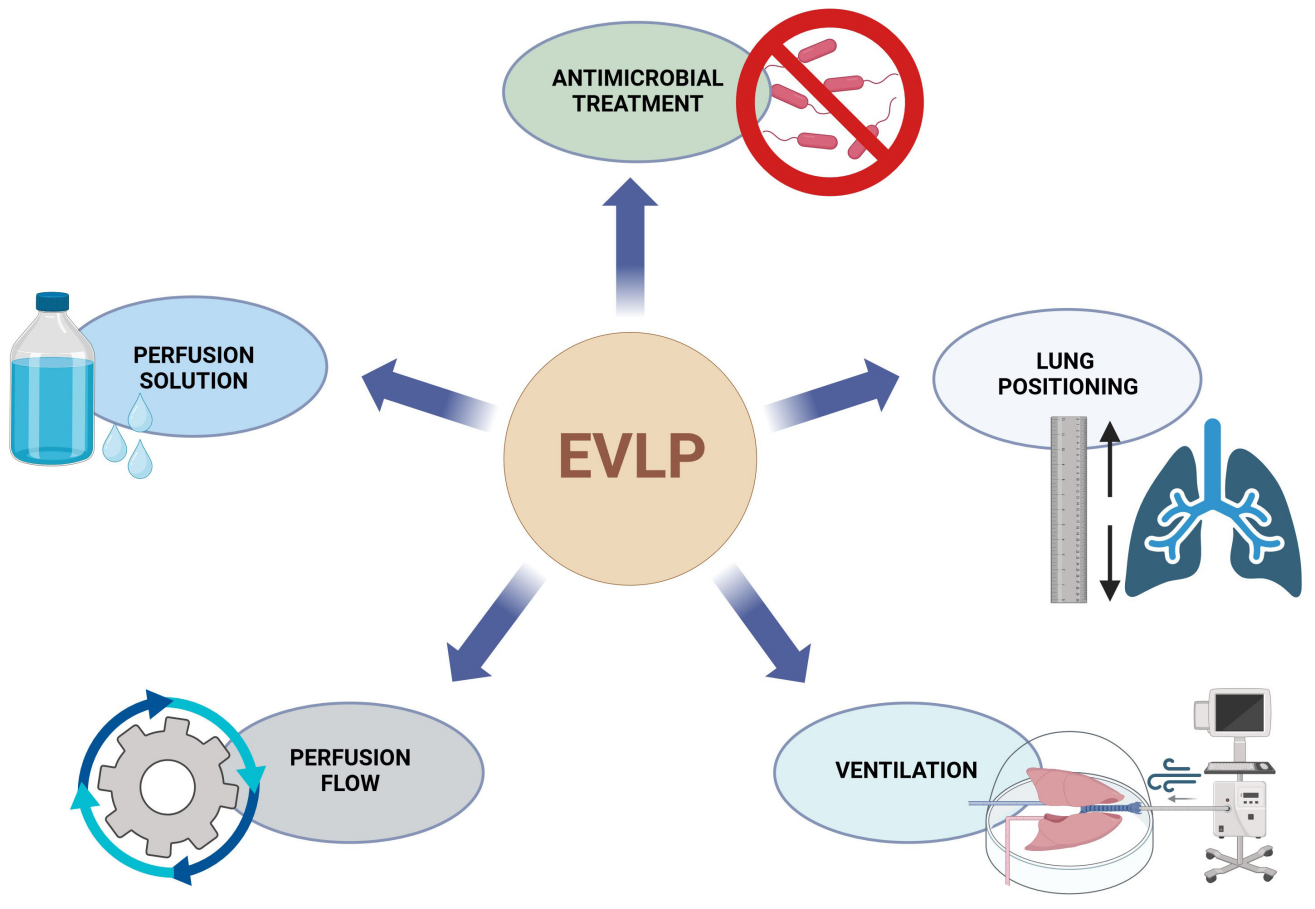
Overview of EVLP and PGD connections. EVLP, ex vivo lung perfusion, PGD, primary graft dysfunction.

At current, experimental, mostly porcine, setups report stable EVLP for up to 72 hours (5, 7). Clinical implementation of these prolonged protocols is still lacking. Previously described interventions for prolongation of stable EVLP include the optimization of the perfusate by adding nutritional factors and dextran, positioning maneuvers, negative pressure ventilation, perfusion pressure/volume or modifying the perfusion temperature and other (**Figure 1**) (8–12).

### 1.1 Mechanisms of action

Ischemia reperfusion injury (IRI) is a process induced by retrieval, storage and transplantation. In contrast to other solid organs, hypoxia is a reduced driving force amongst the mechanisms leading to injury since the lungs remain ventilated during the retrieval process and are cold stored in an inflated, but ischemic state. Hence tissue oxygenation is maintained to a certain extend. Nevertheless, the interruption of blood flow induces depolarizing mechanotransductional effects. This sparks production of reactive oxygen species (ROS) via the activation of NADPH oxidase and NO synthase eventually resulting in vasodilatation and angiogenesis (13). ROS can induce hypoxia-inducible factor 1α (HIF-1α) under normoxic conditions, which further promotes lung edema formation by inducing vascular endothelial growth factor (VEGF) (14–16). Mediated through this cascade, prolonged cold ischemia times leads to activation of necroptosis pathways and inflammatory cell infiltration (17). Nevertheless, literature is lacking data regarding anti-VEGF treatment during EVLP to reduce IRI.

Following reperfusion, oxidant production and complement activation is induced (18, 19). As a result, excess ROS production in the electron transport chain, as key factors in IRI, is prompted (20–22). With rising oxygen levels, also increased ROS production is triggered (18, 23). ROS cause oxidative stress and cell damage, death and inflammation through damage to nucleic acids, proteins and lipids. This damage activates apoptosis and necrosis and triggers an immune response through endogenous damage-associated molecular pattern molecules (23, 24). At the onset of apoptosis the redistribution of phosphatidylserine and loss of anticoagulant surface factors leads to increasing procoagulant tendencies in the endothelial cells (25). Hypoxia further causes a rise in plasminogen activator inhibitor-1 (PAI-1), which is associated with increased pulmonary fibrin deposition, intravascular fibrin and 125I-fibrinogen/fibrin. These mechanisms advance hypoxia-induced thrombosis and form a procoagulant and blood flow restricting milieu (26, 27).

**Figure 2:**
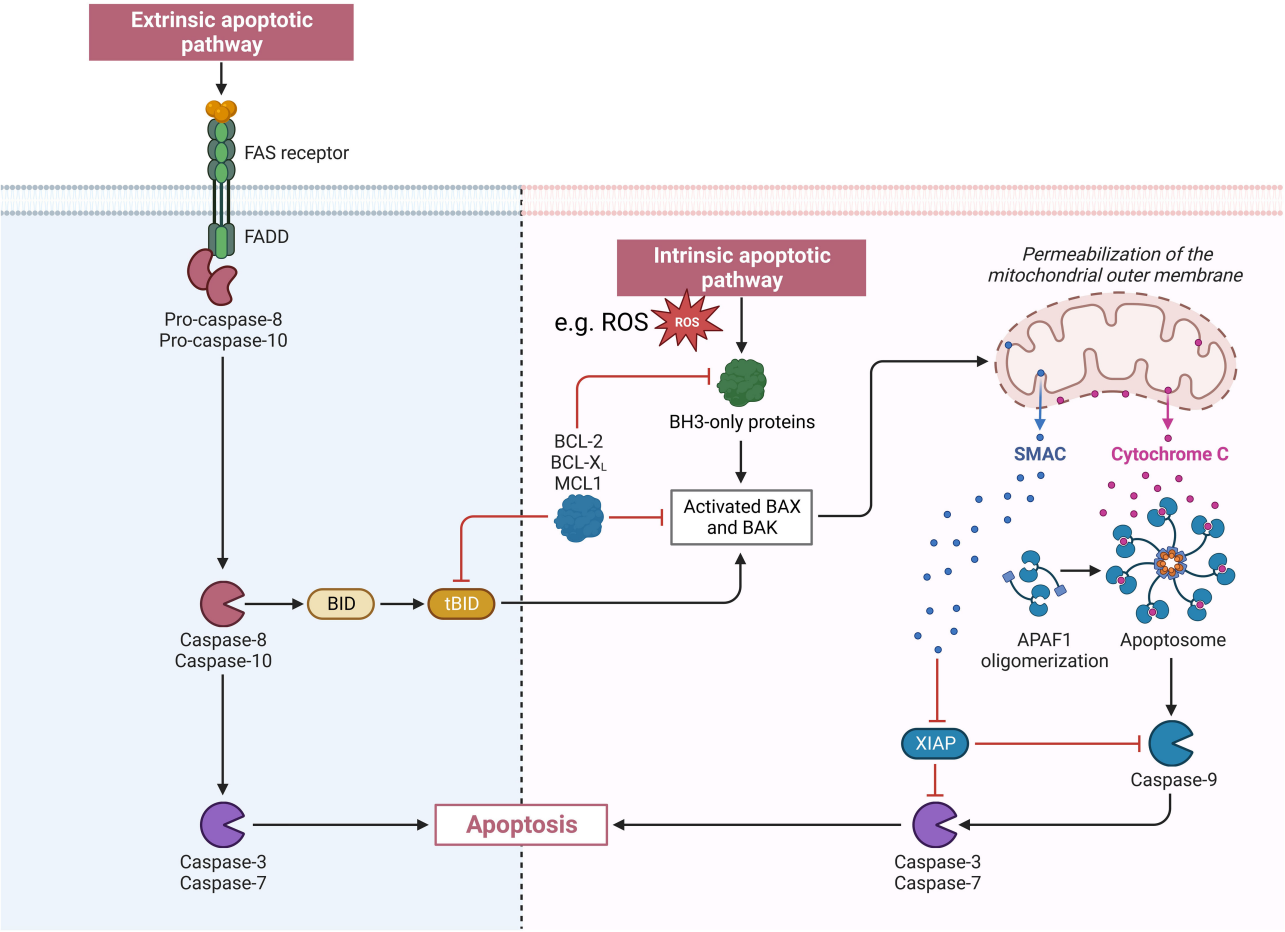
Visualization of the extrinsic and intrinsic apoptosis pathway. APAF1, apoptotic peptidase activating factor 1; BAK, BCL2 antagonist/killer 1; BAX, BCL2 associated X; BCL-2, BCL2 apoptosis regulator; BCL-XL, B-cell lymphoma-extra large (encoded by the BCL2 like 1 gene); BH3, BCL2 homology region 3; BID, BH3 interacting domain death antagonist; FADD, Fas associated via death domain; FAS receptor, Fas cell surface death receptor; MCL 1, MCL1 apoptosis regulator; ROS, reactive oxygen species; SMAC, second mitochondria-derived activator of caspase; tBID, truncated BID; XIAP, X-linked inhibitor of apoptosis.

Apoptosis can be induced via an extrinsic or an intrinsic pathway through caspase 8, or caspase 9. Extrinsic apoptosis is mediated through tumor necrosis factor alpha receptors and intrinsic apoptosis through intracellular mechanisms, such as oxidants or genotoxic effects (28). For intrinsic apoptosis Bcl-2 family proteins stabilize the outer membrane of mitochondria to keep cytochrome c in the cristae. Nevertheless, Bcl-2 proteins include proapoptotic proteins like Bax and Bac too. Bax and Bac lead to increased permeabilization of the outer membrane of the mitochondria. This starts a destabilizing cascade on the inner membrane of the mitochondria leading to a release of cytochrome c. If a specific threshold of cytochrome c is released, through damage to a large amount of mitochondria, apoptosis follows (29, 30). After binding to apoptotic protease-activating factor 1 apoptosomes are formed, activating procaspase 9 (31, 32). At the end of both induction pathways the executioner caspases 3 and 7 are activated (28). An overview of the extrinsic and intrinsic apoptosis pathway is shown in **Figure 2**.

During hypoxia adenosine triphosphate (ATP) decreases and leads to accumulation of hypoxanthine (33). Fisher et al. report of a threshold of 7 mmHg or lower partial pressure of oxygen in the alveoli for this decrease in ATP to take place (34). Independently from ischemia, xanthine dehydrogenase is distributed in many tissues such as the lungs and liver. During ischemia xanthine dehydrogenase is increasingly converted to xanthine oxidase. When the partial pressure of oxygen rises again after reperfusion/reventilation, xanthine oxidase oxidizes the accumulated hypoxanthine and results in a burst of superoxide and hydrogen peroxide (35–37). Accompanying hypoxia seems to increase the activity of xanthine oxidase, while hyperoxia inversely influences activity levels (38). A rat model by Maia et al. proposes that xanthine dehydrogenase might be an even more efficient producer of superoxide when (hypo-)xanthine oxidation is induced (39). Additionally, interferon gamma, upregulated via interleukin-12 and interleukin 18, induces xanthine dehydrogenase and oxidase activity (40, 41).

**Figure 3:**
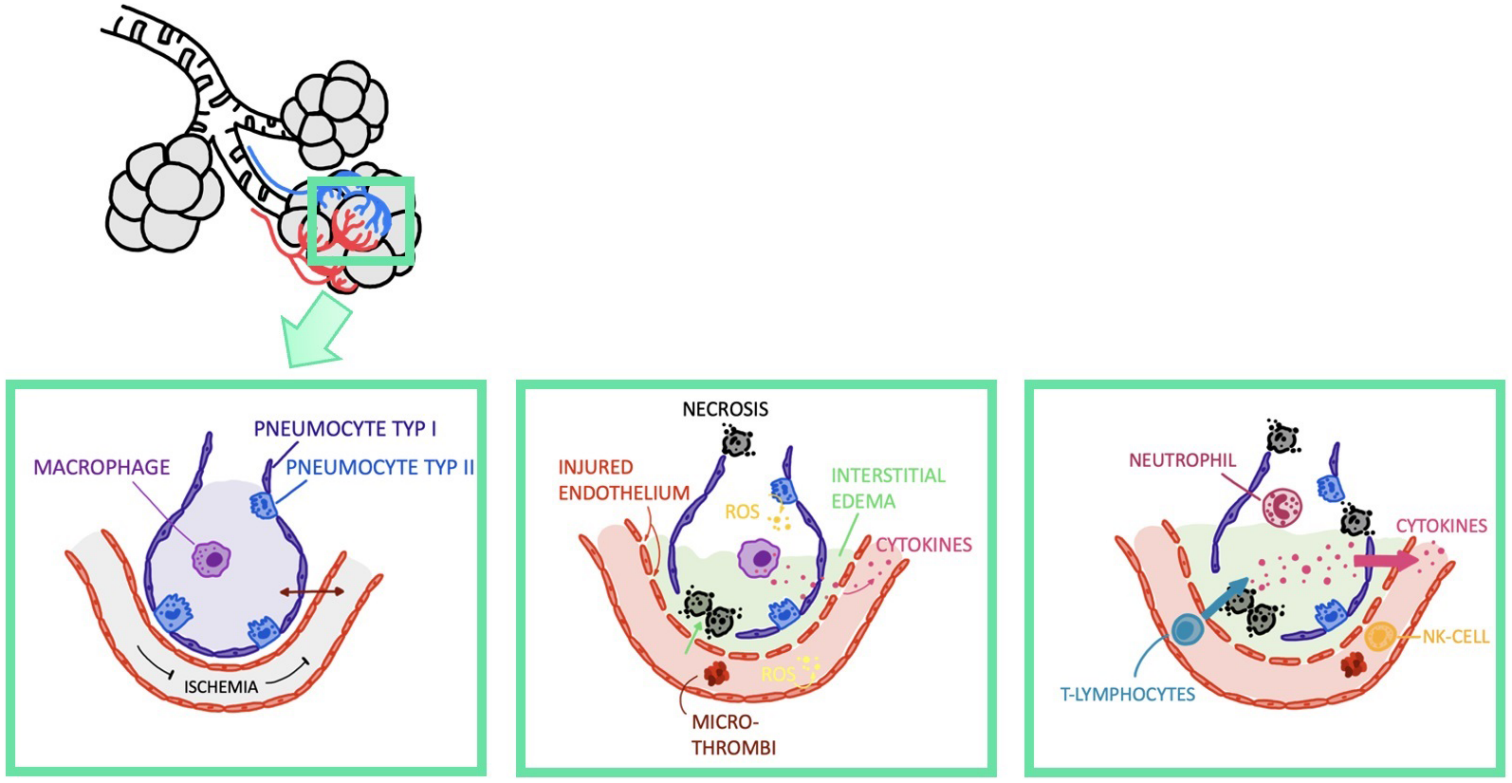
Visualization of ischemia reperfusion injury. NK-Cell, Natural Killer Cell.

A schematic outline of IRI is shown in **Figure 3**. PGD occurs as a result of IRI and negatively impacts the outcome. Even if PGD is treated successfully, patients are at a higher risk for developing Bronchiolitis obliterans syndrome (BOS) and face worse long-term lung function (42). Further factors influencing the development of IRI are described in the sections below.

## 2 EVLP methodology and optimization

**Figure 4:**
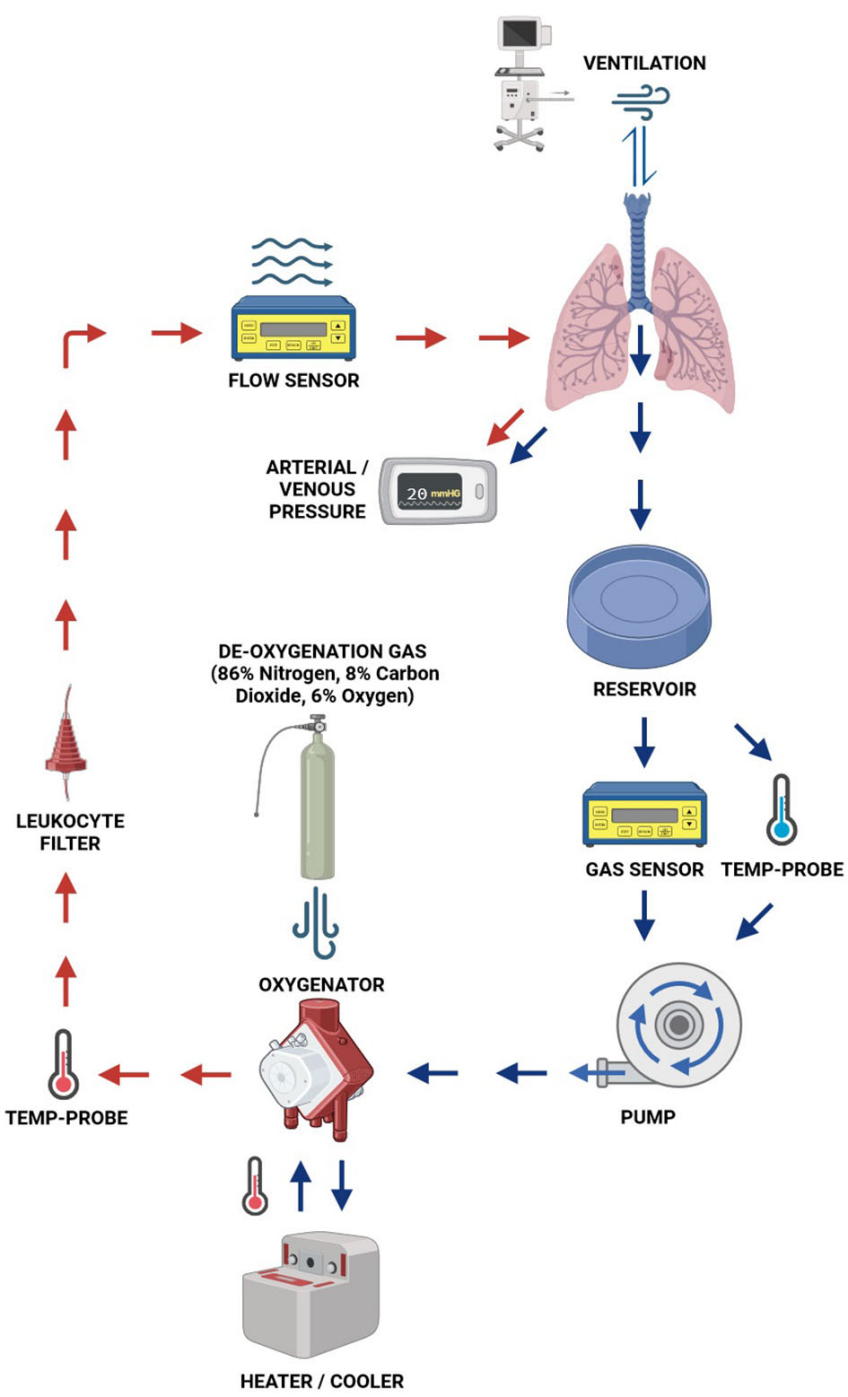
Basic EVLP setup.

Since its first clinical use in 2000 in Lund (Sweden), various commercially available EVLP systems have been developed (43). Most of the systems apply similar basic principles (**Figure 4**) and protocols described as the Lund, Toronto or the Organ Care System (OCS™) protocol. Currently commercially available systems include, but are not limited to, the OCS (Transmedics, Andover, MA, USA), XVIVO Perfusion System (XPS™,XVIVO Perfusion AB, Göteborg, Sweden) and TorEx Lung Perfusion System (Traferox,Technologies Inc., Mississauga, Canada).

**Figure 5:**
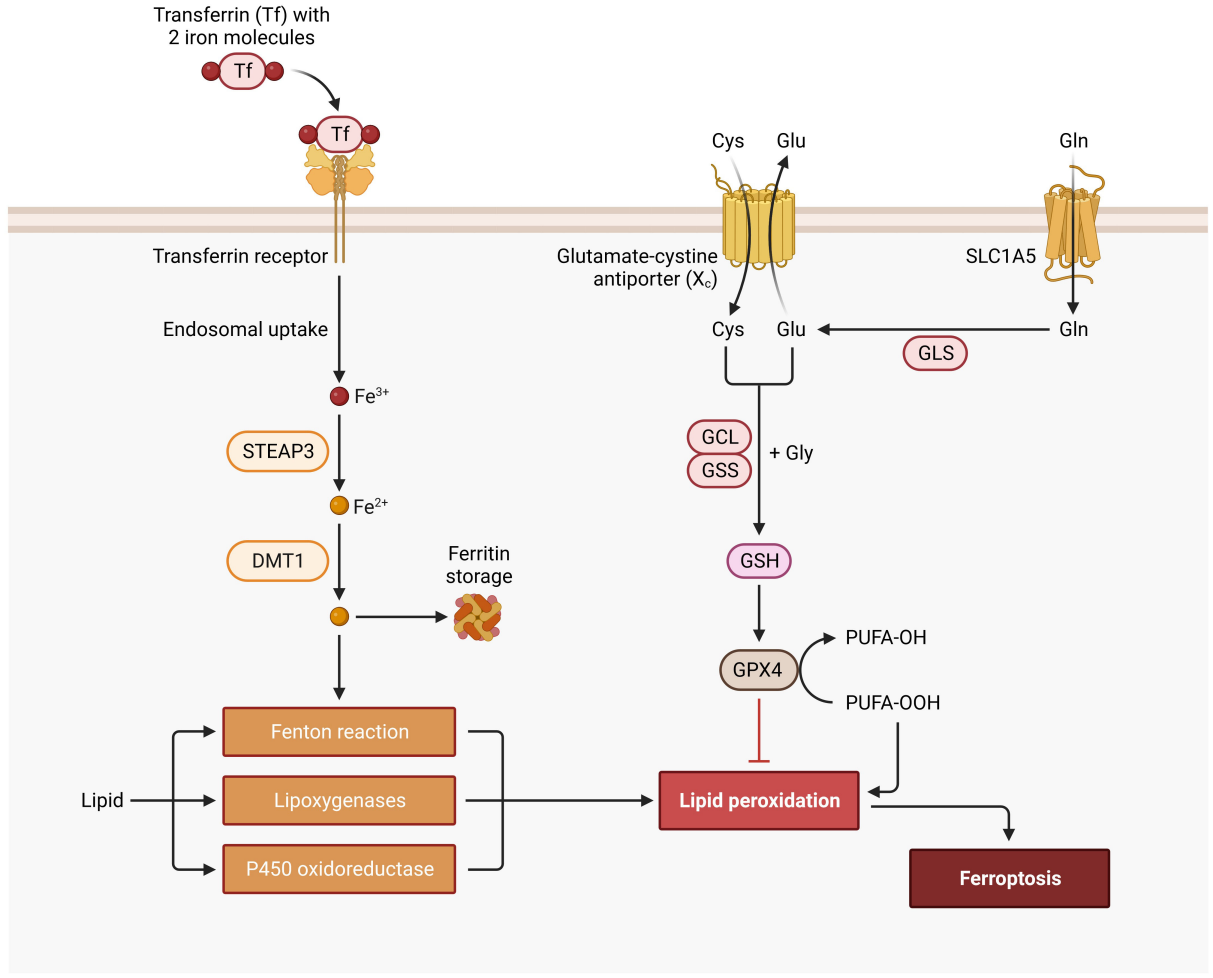
Ferroptosis Signaling Pathway. Cys, cysteine; DMT1, divalent metal transporter 1; FE, iron; GCL, glutamate-cysteine ligase; Gln, glutamine; GLS, glutaminase; Glu, glutamate; Gly, glycine; GPX4, glutathione peroxidase 4; GSH, glutathione; GSS, glutathione synthetase; PUFA, polyunsaturated fatty acid; SLC1A5, solute carrier family 1 member 5; STEAP3, six-transmembrane epithelial antigen of prostate.

Systems vary in their ventilation and perfusion setting options and their respective recommended protocols. The XVIVO XPS and TorEx both use the Toronto protocol while for the OCS a separate protocol is recommended. The OCS system operates with an open circuit, meaning that no atrial cuff is used, and uses a pulsatile pump in comparison to a continuous one. An advantage of the XVIVO XPS represents its organ chamber design, which allows for easy radiographic imaging. Publicly available information for the TorEx Lung Perfusion System is scarce except that it is based on the Toronto protocol and is considered a plug-and-play system, which simplifies the setup.

These protocols vary in perfusate, perfusion parameters and ventilation. An overview can be seen in **Table 1**.

**Table 1:** Overview of clinically used EVLP protocols (**44**–**46**).

	Lund	Toronto	OCS ™
Perfusion Solution	STEEN Solution ™ with added red cell concentrates	STEEN Solution ™	OCS ™ Lung Solution with added red cell concentrates
Perfusion Flow	100% of cardiac output	40% of cardiac output	2-2.5 l/min
Pulmonary Arterial Pressure (mmHg)	≤20	≤15	≤20
Center Atrial Pressure (mmHg)	O (no atrial cuff)	3-5	0 (no atrial cuff)
Mode of Perfusion Flow	Continuous	Continuous	Pulsatile
Mode of Ventilation	Volume Controlled	Volume Controlled	Volume Controlled
Tidal Volume (ml/kg)	6-8	7	6
Peak End-Expiratory Pressure (cmH2O)	5	5	5
Fraction of Inspired O2 (%)	50	21	21
Ventilation Frequency (bpm)	10-15	7	10
Temperature at Start of Ventilation (°C)	32	32	32
Inflow Temperature of Perfusate at Start of Perfusion (°C)	15	25	32
Temperature at Start of Assessment (°C)	37	37	37

Adapted from Andreasson et al. (47).

### 2.1 Perfusion solution

There are two types of perfusion solutions for EVLP: cellular and acellular. The Toronto protocol uses an acellular perfusate, while the Lund and OCS protocol utilize RBCs (47). The Lund and Toronto protocol both use STEEN Solution™ as a basis (XVIVO Perfusion, Goteborg, Sweden), while the Lund protocol employs red blood cell concentrates. The OCS is approved to be used with the OCS™ Lung Solution (Transmedics, Andover, MA, USA) with added red blood cell concentrates (44–46). The outcome of the respective studies comparing different perfusion solutions remains inconclusive (48–50).

Perfusion solutions may have a significant impact on ROS production making them a viable point for optimization. A promising option is to reduce cell-damage by introducing iron chelators with the perfusion solution, such as Custodiol-N or Custodiol-MP (Dr. F. Köhler Chemie, Bensheim, Germany). This has proven to significantly reduce ROS production and improve functional parameters (18, 51, 52). Ferroptosis leads to iron-dependent programmed cell death through lipid peroxidation (**Figure 5**). In ferroptosis the outer membrane of the mitochondria fragments while the cristae disappear. The pathway starts with iron entering the cell through transferrin receptors. After disintegration from endosomes iron is stored in the labile iron pool. An overload of the labile iron pool together with hydrogen peroxide leads to ROS production through the Fenton reaction. ROS is responsible for lipid peroxidation, because of reacting with polyunsaturated fatty acids of lipid membranes, which ultimately leads to ferroptosis (53, 54). An inhibitory pathway of ferroptosis is initiated with cystine entering the cell through cystine/glutamate reverse transporters. Afterwards it is reduced to cysteine. Glutamate-cysteine ligase and glutathione synthetase synthesize glutathione with the use of cysteine, glutamic acid and glycine. Glutathione then acts as a cofactor for glutathione peroxidase 4, which reduces lipid peroxidation and suppresses ferroptosis (54–56). These ferroptosis pathways can be targeted with iron chelators (e.g. deferoxamine) through a reduction of the iron load (53).

**Figure 6:**
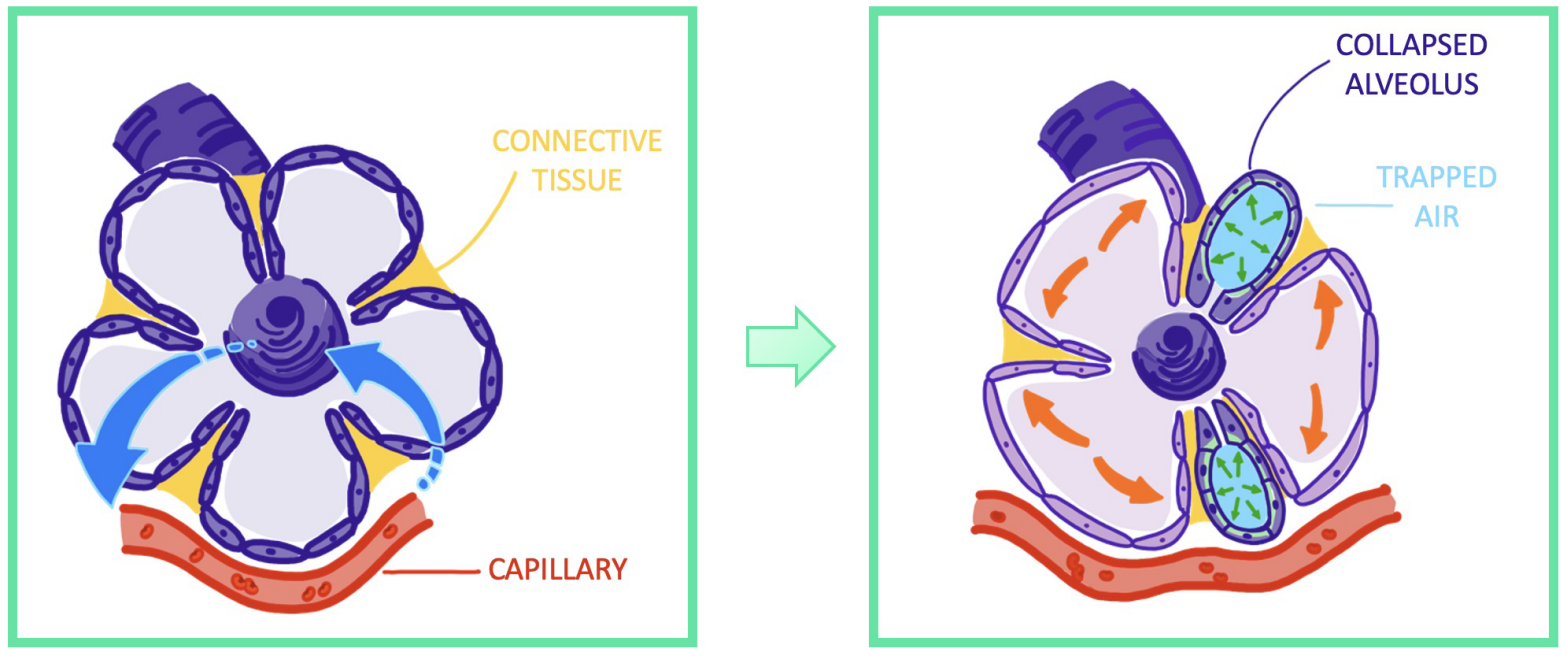
Visualization of atelectrauma through repeated recruitment and derecruitement of functional lung airway units.

RBC-based perfusates offer the advantage of assessing and perfusing the lungs under more physiological conditions. The addition of erythrocytes to the perfusate leads to better oxygen binding capacity and a more precise evaluation of the oxygenation capability. Acellular perfusion might lead to a flawed assessment of oxygenation capability using blood gas analysis as shunting during ex vivo perfusion might have reduced effects on the measured partial pressure of oxygen as a result of linear curve of oxygen content and partial pressure of oxygen (in comparison to the sigmoidal relationship for perfusates with hemoglobin) (57–59). This is of utmost importance, since blood gas analysis is a key determining factor during EVLP for predicting the outcome. Conversely, RBC based perfusates hold the risk of hemolysis during EVLP. Because of the mechanical stress towards RBCs through pumping of the perfusate, red blood cells may rupture and become dysfunctional or irreversibly stiffened. Thus the increased physical resistance may lead to consecutive mechanical lung injury (44, 60–62).

Further to functional aspects, RBC-based perfusion requires more personnel because of subsequently rising demand for blood concentrates. This also further aggravates costs related to EVLP and might deter from using EVLP with a RBC-based perfusate. Additionally, blood concentrates might be scarce or not available at all leading to a possible ethical dilemma for research protocols using cellular perfusates. For clinical settings alternative perfusion solutions should be in place to avoid unsuccessful EVLP due to constrained blood concentrate availability.

Recent data by Olbertz et al. suggest that using Custodiol-N with added glucose monohydrate, dextran 40 and albumin helps stabilizing lung function through a higher oxygen capacity and lower wet-to-dry ratio in comparison to STEEN Solution™. Moreover, Olbertz et al. report a lower peak airway pressures when using the above-mentioned perfusate (51). The same group also showed a significantly better oxygenation capacity, lower lactate dehydrogenase activity and lower lactate concentrations when using Custodiol-MP in a porcine EVLP model (52) (**Table 2**).

**Table 2:** Composition of modified Custodiol-N, Custodiol-MP base perfusion solution, Custodiol-MP final perfusion solution and Steen Solution™.

	Modified Custodiol-N (mmol/L)	Steen Solution™ (mmol/L)	Custodiol-MP Base Solution	Custodiol-MP Final Solution*
Sodium	16	86	18.8	~47
Potassium	10	4.6	8.4	5.8
Magnesium	8	0.8	11.5	8.0
Calcium	0.02	1.5	0.06	0.04
Chloride	30		26.2	~<45
L-Histidine	124		110.0	76.5
N-α-Acetyl-L-Histidine	57		77.4	53.8
Tryptophan	2		2.6	1.8
α-Ketoglutarate	2		1.6	1.1
Aspartate	5		4.2	2.9
Arginine	3		4.5	3.1
Alanine	5		8.4	5.8
Glycine	10		14.6	10.2
Sucrose	33		41.8	29.1
Deferoxamine	0.025			0.025
LK-614	0.0075			0.0075
Pyruvate			4.2	2.9
Caprylate				4.4
N-Acetyltryptophan				4.4
Dextran 40 (g/l)	50	5		
Albumin (g/l)	7	70		54.5
Glucose	-	11		8.8
Phosphate	-	1.2	0.6	0.4
pH	7.0	7.4 adjusted	7.0	7.0
Osmolarity (mosm/l)	306		335	<312

Values in mmol/L.

*including lyophilisate, 35 ml 5% glucose solution, 300 ml human albumin 20% (CLS Behring GmbH, Marburg, Germany).

Data taken from Olbertz et al., Kniepeiss et al. and Kalka et al. (51, 52, 63).

Huang et al. proposed a newly designed perfusate based on Dulbecco’s Modified Eagle Medium, containing essential and non-essential amino acids and specific vitamins, in combination with 5 g/L dextran 40 and 7% albumin. This perfusate (“D05D7A”) showed improved cell confluence, reduced apoptosis and better migration in a cell culture based study when compared to Steen Solution. D05D7A also lead to higher glutathione production, which is a key factor in inhibition of ferroptosis. Interestingly, the low apoptosis rate in the D05D7A group could be sustained for continuous 48h, which provides a possibility for further stabilizing prolonged EVLP protocols (64). A trial of this solution, outside of culture based protocols, is still missing.

The use of cytokine adsorption filters during EVLP might further mitigate pro-inflammatory effects of cytokines. Various studies have shown the positive effects of cytokine filtration on pulmonary edema development, microscopic lung injury, inflammatory response, peak airway pressure and decreased pro-inflammatory cytokines (65–68). Boffini et al. even report of reduced in-hospital mortality and 1-year death rate in a cytokine adsorption cohort when retrospectively comparing their EVLP patients. Yet it has to be noted, that the cohort without cytokine adsorption was in the beginning of their EVLP program and required cardiopulmonary bypass during transplantation more often (68).

In conclusion, the optimal perfusate remains unclear. Considering above-mentioned aspects, the use of acellular perfusates in prolonged EVLP might be advantageous. This might avoid detrimental effects of hemolysis and mechanical lung injury, which cumulate over time during EVLP. For shorter perfusion times and routine clinical use, RBC based perfusates may offer an advantage for evaluation of the donor lungs.

### 2.2 Perfusion characteristics

EVLP protocols differ with respect to flow rate and flow pattern. Favorable results have been shown for certain settings. Flow rates in clinical EVLP protocols range from fixed values (2 - 2.5 l/min) to 40 - 100% of cardiac output (47). Perfusion volume per minute is highest in the Lund protocol with a flow of 100% of cardiac output. The Toronto protocol applies 40% of cardiac output. Both protocols initiate EVLP with lower than targeted flow rates and gradually increase flow while temperature increases to 37°C (12, 69, 70). The OCS protocol predetermines the flow rate at 2 and 2.5 l/min and also gradually increases flow from start of EVLP until reaching the target temperature of 37°C (70–72).

Beller et al. suggest a low flow protocol with only 20% of predicted cardiac output. They have shown significantly higher pulmonary pO2, compliance, reduced lung wet-to-dry ratio and lower IL-1β (73). These low flow protocols were already successfully used in a rat and in a porcine model (73, 74). The improved results may be attributed to the reduced vascular stress and subsequent inflammation and fluid permeability (47, 73, 74).

The pulmonary arterial pressure (PAP) should not exceed 20 mm Hg perfusion pressure in both the Lund and OCS protocol, and 15 mmHg PAP in the Toronto protocol (11, 44, 47, 69, 75, 76). Upper limits for perfusion pressure are important for clinical evaluation of donor lungs since a higher PAP corresponds with impaired lung function and possible edema formation. Various study groups examined flow mechanics on EVLP and confirmed this finding (73, 77, 78).

Management of the left atrium further influences the vascular behavior and flow dynamics. The Lund and OCS protocol both use an open system, while the Toronto protocol attaches a cuff to the left atrium. With this cuff, the left atrial pressure is kept at about 3-5 mm Hg. Linacre et al. show that an open circuit in EVLP results in a lower success rate in prolonged EVLP because of worse oxygenation, decreased lung compliance, increased vascular resistance and peak inspiratory pressure. Consistent with these findings, the wet-to-dry ratio and lung edema score was worse in the open group. Linacre et al. hypothesize that this difference might be due to the missing venous afterload pressure with a subsequent change in lung perfusion zones and *“a cyclical open-close phenomenon at the capillary level with ventilation exacerbated at low after-load pressures and leading to endothelial cell injury, vascular dysfunction, alteration of permeability and edema formation”* (78). This may amplify the proinflammatory and procoagulant hypoxia-induced changes through translocation of phosphatidylserine, stimulating activated platelet adhesion (27). Phosphatidylserine offers a possibility for targeted therapy. Diannexin is a homodimer of Annexin V, which binds to phosphatidylserine and inhibits prothrombinase complexes and secretory phospholipase A2. Consequently, hydrolyzation of cells’ phospholipids is inhibited and reduces coagulation (79). Data from a murine transplant study showed lower wet-to-dry ratio, higher partial pressure of oxygen, lower alveolar fibrin deposition score and reduced caspase-cleaved cytokeratin 18, which acts as a marker for apoptosis (27). Clinical data of its usage as a prophylactic IRI therapy in EVLP is still lacking.

Furthermore, flow characteristics can be divided in continuous and pulsatile flow. Most EVLP devices use continuous flow, while the OCS Lung (Transmedics, Andover, MA, USA) applies pulsatile flow generated by a piston pump (47, 80). Even though effects of pulsatile flow on functional outcome parameters such as inflammation or pulmonary edema are still unclear, it might provide a more physiological setting for evaluation (80, 81).

### 2.3 Ventilation

During EVLP, donor lungs are mechanically ventilated to assess and preserve their function. Ventilator settings during EVLP are similar to conventional ventilation. However, mechanical ventilation (MVe) with positive pressure bares the risk of ventilator induced lung injury (VILI). VILI may be induced in the donor and prior to organ retrieval and and/or during EVLP MVe. The driving pressure during EVLP MVe is a result of various factors, which are dependent on the lungs and surrounding tissue, but also influenced by ventilator settings, including: flow at the tracheal opening, lung resistance (LE), lung elastance, and lung volume, which is relative to the functional residual capacity (82, 83). The most important and modifiable parameter is LE.

Volutrauma and atelectrauma are the two main stressors triggering LE and progression of VILI. Barotrauma does not seem to be relevant for the development of VILI. Data generated by Dreyfuss et al. suggest that the increased inflation volume through high pressures is responsible for tissue damage. Rat lungs ventilated with high tidal volume ventilation showed significantly increased edema development while high pressure ventilation did not (84). *In-situ*, damage through high driving pressure may be reduced as the thoracic cavity provides some natural resistance against massive distension of the lungs. With EVLP, this natural resistance is completely lost. Therefore, optimal settings of EVLP MVe need to focus on tidal volume and associated driving pressure.

Atelectrauma is the result of multiple opening and closing events of lung airway units, which are caused by recruitment of these units during inspiration and derecruitement during expiration. Emerging shear forces injure the epithelium and lead to epithelial necrosis, as is depicted in **Figure 6**.

To mitigate the risk of atelectrauma optimal positive end-expiratory pressure (PEEP) settings are warranted in order to reduce the number of recruitment cycles and keep lung units open (83, 85, 86). Therefore, all three clinical EVLP protocols utilize a volume-controlled ventilation mode with settings between 6 and 8 ml/kg donor weight. PEEP is kept at 5cmH2O in all protocols (47). With these settings, the dynamics of the driving pressure to achieve target volumes have merit as functional parameters for lung assessment.

Recent data suggest, that flow-controlled ventilation (FCV) may further reduce VILI. In flow-controlled ventilation, the expiration is linear, the inspiration/expiration-ratio equal and independent of recoil forces of the lung. Through this ventilation, the amount of dissipated energy during ventilation cycles is minimized and lung tissue damage prevented (87, 88). Moreover, FCV improves oxygenation and carbon dioxide elimination during MVe (89). Goebel et al. reported increased lung compliance, lower PEEP and lower wet-to-dry ratios in a porcine model (88). These results are reproducible in EVLP. Ordies et al. show that in a porcine EVLP model, FCV provides significantly better oxygenation and improved lung compliance in a larger area of ventilated lung tissue. Despite these superior functional results, total lung injury did not differ in comparison to volume controlled ventilation (90).

Another promising method is negative pressure ventilation (NPV) in which inspiration is modelled by placing the donor lungs in an air-tight chamber and applying negative pressure. This method does not rely on the force of positive pressure ventilation and might therefore reduce the risks of physical damage to the lung. Supporting evidence for this concept was generated in an EVLP model. Better compliance, reduced pulmonary vascular resistance and reduced weight gain during EVLP were found, while oxygenation remained unchanged (91). NPV is associated with reduced secretion of proinflammatory cytokines, such as tumor necrosis factor alpha, interleukin-6, and interleukin-8. Noteworthy, Aboelnazar et al. reported weight reduction and lower lung injury through NPV (92). Buchko et al. tested NPV in a clinical trial with 12 LuTx patients. They have established feasibility and demonstrated good outcomes with a PGD grade 0 rate at 72 hours of 83% (grade 1: 0%, grade 2: 17%, grade 3: 0%) (93).

The fraction of oxygen (FiO2) is set at 21% for the Toronto and OCS protocol and at 50% for the Lund protocol (47, 70). Various randomized trials are examining higher vs. lower FiO2 in ventilated patients on intensive care units displaying a trend towards an increase in mortality and serious adverse event, but the effects of higher FiO2 in EVLP remain unknown (94). To our knowledge, no studies addressing the influence of FiO2 in EVLP are available. In theory, higher FiO2 may lead to hyperoxemia and hyperoxia, which results in increased ROS (hydroxyl radicals and superoxide ions) production. This effect is evident in EVLP, since IRI is induced – albeit in a different form. Furthermore, higher FiO2 causes an alveolar nitrogen washout, which is subsequently responsible for atelectasis through the dislocation of oxygen from the alveoli to capillaries (23). Further studies are needed to assess the relevance of different FiO2 strategies in EVLP.

**Figure 7:**
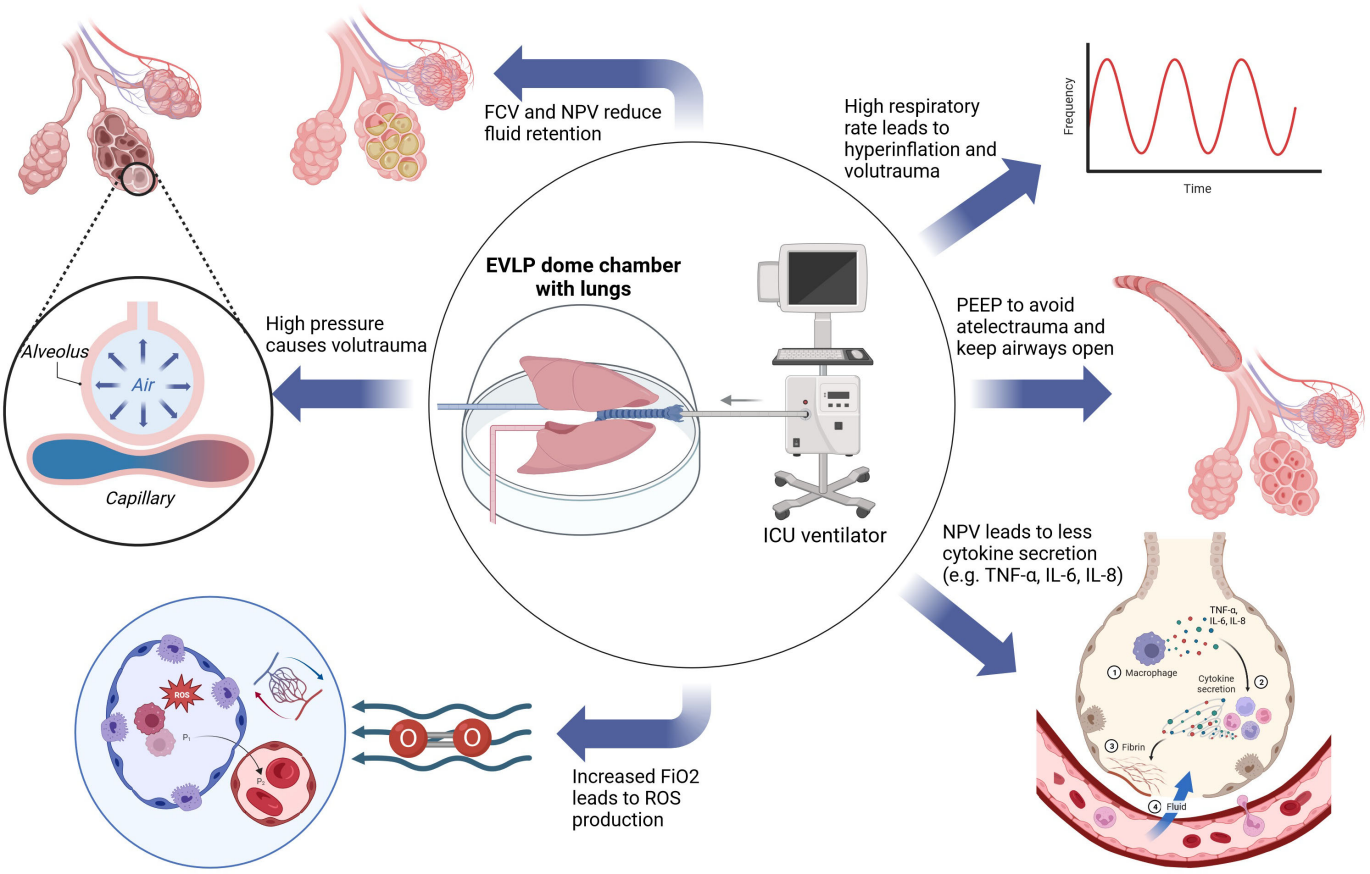
Aspects of the influence of ventilation during EVLP on the lungs. EVLP, ex vivo lung perfusion; FCV, flow-controlled ventilation; FiO2, fraction of inspired oxygen; ICU, intensive care unit; IL-6, interleukin 6; IL-8, interleukin 8; NPV, negative pressure ventilation; PEEP, positive end expiratory pressure; TNF-α, tumor necrosis factor-α; ROS, reactive oxygen species.

Respiratory rate is set at seven breaths per minute (bpm) in the Toronto protocol, 10 bpm in the OCS protocol and 10-15 bpm in the Lund protocol (47, 70). The standard MVe respiratory rate is 12-16 bpm and dynamically adjusted accordingly to achieve eucapnia. The respiratory rate may be increased to avoid hypercapnia or to compensate acidosis. Each increase of the respiratory rate, however, poses the risk of dynamic hyperinflation and volutrauma in donor lungs (95, 96). Specific data regarding the respiratory rate during EVLP are not available, but an extrapolation from *in-vivo* data may help. In an analysis of 102.632 cases, Santer et al. report that an increased respiratory rate (median 8 vs. 15 bpm) was associated with a significantly higher rate of postoperative respiratory complications and postoperative healthcare utilization (97). All three protocols use respiratory settings in or below the physiological range, but a direct comparison has not been performed. An overview of the influence of different ventilation modification on the lung during EVLP is visualized in **Figure 7**.

### 2.4 Lung positioning

As primary graft dysfunction (PGD) is associated with elevated levels of interstitial fluid retention, gravitational forces should be considered. Conceptually, lungs could be treated as healthy lungs or rather as borderline intensive care unit patients. The typical EVLP setup places the donor lungs in a supine position. For treatment of acute respiratory distress syndrome (ARDS) and acute lung injury, prone positioning has shown to reduce mortality and positively impact oxygenation. Complete avoidance of edema formation during EVLP or ARDS seems difficult, but optimal management may help to reduce its negative impact. When edema is forming, the pressure of the superimposed lung parenchyma leads to a collapse of the surrounding parenchyma and impact organ function. Placing the lungs in a prone position helps to recover the dorsal regions through changing the direction of gravitational forces and respective hydrostatic pressure (98, 99). Ordies et al. demonstrated, that prone EVLP leads to a more homogenous distribution of pulmonary edema (100). The dorsal regions possess a higher volume of lung parenchyma, which may explain this observation.

Findings in EVLP confirmed this effect. Prone position EVLP improves partial pressure of oxygen/fraction of inspired oxygen ratio (P/F) and compliance. Interestingly, prone position reduces the amount of inflammatory cytokine expression (interleukin-1β, interleukin-8, tissue tumor necrosis factor alpha) in the lower lobes, while the interleukin-10, which has an anti-inflammatory effect through inhibition of macrophages or tissue tumor necrosis factor alpha among others, did not change significantly (100–102). A cytokine profile triggering increased granulocyte recruitment as a result of pro-inflammatory cytokines and reduced inhibition through interleukin-10, might be an important factor for PGD development. Neutrophils are capable of releasing ROS and neutrophil extracellular traps (NET). Through increased ROS production, NETosis increases and leads to further aggravation of inflammation and edema (103–105). The impact on lung injury and associated PGD was confirmed in a murine PGD model. In this study, NET formation and PGD development could be reduced with administration of acetylsalicylic acid and disrupted with direct intrabronchial administration of DNaseI (106).

Studies with human lungs demonstrate the benefit of prone position during EVLP. Niikawa et al. showed that three out of five human lungs, which were rejected for clinical use, could have been suitable for transplant after prone EVLP while zero out of five lungs placed in the supine position fulfilled the criteria for transplantation (102). Niikawa et al. also demonstrated the feasibility of prone EVLP in a case report, in which one of two human prone EVLP lungs, both with reduced lung edema after EVLP, was successfully transplanted (107).

### 2.5 Antimicrobial treatment

During EVLP, donor lungs may be susceptible for colonization of microbial organisms. Contamination during organ retrieval is not uncommon and colonization during EVLP with massive germ transmission during transplantation represents a realistic threat. Heavy immunosuppression upon transplantation further aggravates this situation and makes is potentially life threatening. Aguilar-Guisado et al. reported an incidence of 72 episodes of post-transplant pneumonia per 100 LuTx. Bacterial infections were the most common cause (82.7%) (108). Colonization in the donor was responsible for 7.6% recipient infections (109). Such infections negatively affect short- and long-term outcome in LuTx patients (110). Andreasson et al. demonstrated that antimicrobial treatment with a single-dose of Meropenem (500mg) at the start of EVLP significantly reduces bacterial load after EVLP. Moreover, the yeast load increased during EVLP without anti-fungal treatment but decreased if Amphotericin B (50mg) was administered at the start of perfusion (110). Data by Nakajima et al. confirmed the importance of antimicrobial treatment during EVLP and the therapeutic possibilities. EVLP lungs, which were treated with Ciprofloxacin or Azithromycin, Vancomycin and Meropenem, showed significantly reduced bacterial counts in BAL, improved pulmonary oxygenation better compliance and reduced vascular resistance (111). These results also provide a cornerstone for EVLP as a therapeutic platform for reconditioning donor lungs with infections for transplantation, which would otherwise be rejected.

Because it is not possible to receive bacterial cultures for targeted anti-microbial therapy in time, treatment mostly remains empirical. Established treatment regimens show promising results, but further refinement of the anti-microbial drug therapy is warranted. Prolonged EVLP requires broad-spectrum anti-microbial treatment to prevent microbial infections and inflammation.

The lung microbiome is thought to play a role in PGD development, acute rejection, bronchiolitis obliterans syndrome and restrictive allograft syndrome (112, 113). Pseudomonas species, especially Pseudomonas aeruginosa (which is sensitive to Meropenem), are related to the development of BOS and inferior outcome (112, 114). Of note, the addition of Azithromycin to the perfusion solution improved survival in early stage BOS treatment and hence should be considered irrespective of its antimicrobial effect. This protective effect has been related to the effect on neutrophilic airway inflammation via decreased lipopolysaccharide stimulated release of Interleukin-8 and granulocyte macrophage colony stimulating factor (115, 116).

Apart from antibiotics application of high-dose (>160 ppm) nitric oxide (NO) may be used to treat infections caused by bacteria and viruses (117, 118). Although low-dose NO is already clinically used for treatment of ARDS its potential side effects such as hospital/ventilator-acquired pneumonia, acute kidney injury and increase in methemoglobin levels impairs prolonged and/or high-dose NO therapy (118–123). EVLP in combination with an acellular perfusate can overcome this obstacle and allows for continuous high-dose NO treatment. Michaelsen et al. showed the feasibility of high-dose inhaled NO for 12 hours in a porcine EVLP model and reported no differences in vascular resistance, static and dynamic compliance, graft oxygenation, peak airway pressure, levels of pro-inflammatory cytokines or edema formation. Methemoglobin stayed in a safe range during acellular EVLP (118). Although regimens with intermittent, in contrast to continuous, high-dose inhaled NO have been successfully clinically tested, they failed to achieve the same results as *in-vitro* studies (117).

## 3 Summary

EVLP is used for clinical and research purposes. It has become a routine procedure in specialized centers. EVLP plays an important role in the assessment of donor lungs and may ameliorate PGD. A plethora of mechanisms contribute to the effect of EVLP such as reduction of interstitial fluid retention and improvement of function.

Future studies assessing physiological lung assessment and determination of the susceptibility for PGD, might consider using pulsatile perfusion with a cellular perfusate at a flow rate similar to cardiac output and an NPV of 21% FiO2 as suitable settings. FiO2 should not be set to higher-than-normal in order to avoid a possible increase in ROS production, which in turn advances IRI (23). The commercially available perfusion systems and the established protocols have their distinct features while a head-to-head comparison is lacking.

Current trends aim at prolonging EVLP, minimizing PGD rates, and reconditioning of donor organs. For these goals to be achieved, a better understanding of edema formation during EVLP and PGD development after LuTx is key. An important goal in this context is the minimization of ROS and pro-inflammatory cytokine production. This can be achieved by altering the perfusate composition, e.g. through adding iron chelators, acetylsalicylic acid or DNaseI (18, 51, 52, 106). A low flow protocol may also contribute to reduce the wet-to-dry ratio and pro-inflammatory cytokine expression (73, 74). MVe settings impact PGD development, most likely through volu- and atelectrauma. FCV improved oxygenation, lung compliance and wet-to-dry ratio (88–90). NPV reduces secretion of pro-inflammatory cytokines and PGD rates in clinical studies (92, 93).

EVLP can be improved by placing the donor lungs in prone position. Prone positioning reduces pro-inflammatory cytokine expression, protects against PGD and improves P/F and compliance (100–106).

Nevertheless, most of these findings lack clinical implementation and testing, apart from porcine or murine models. As a result available protocols remained greatly unchanged over the last period.

No consensus on the EVLP setup exists and different indications might call for different settings. Although ex vivo perfusion of organs is a clinical reality, many questions remain unanswered. Further prolongation of perfusion times without diminishing organ quality is the immediate goal.

## Author contributions

FP: Conceptualization, Data curation, Formal analysis, Investigation, Methodology, Project administration, Resources, Visualization, Writing – original draft, Writing – review & editing. JD: Investigation, Writing – original draft, Writing – review & editing. CK: Investigation, Writing – original draft, Writing – review & editing. AP: Investigation, Writing – original draft, Writing – review & editing. TH: Investigation, Writing – original draft, Writing – review & editing. DW: Investigation, Writing – original draft, Writing – review & editing. FA: Investigation, Writing – original draft, Writing – review & editing, Conceptualization, Methodology, Project administration, Supervision. SS: Conceptualization, Investigation, Methodology, Project administration, Supervision, Writing – original draft, Writing – review & editing, Resources.
